# Aspirin Desensitization in the Course of Non-ST-Segment Elevation Acute Coronary Syndrome: A Case Report and Review of the Literature

**DOI:** 10.7759/cureus.77365

**Published:** 2025-01-13

**Authors:** Hicham Faliouni, Lalla Ghita Alaoui, Youssef Fihri, Mehdi Laaroussi, Mouad Lamtai, Zouhair Lakhal, Aatif Benyass

**Affiliations:** 1 Cardiology Center, Cardiac Catheterization Unit, Military Hospital Mohamed V, Rabat, MAR; 2 Cardiology, CHU (Centre Hospitalo-Universitaire) Ibn Sina, Rabat, MAR

**Keywords:** angioplasty, aspirin, dapt, desensitization, nstemi

## Abstract

Antiplatelet therapy is essential for patients with acute coronary syndrome (ACS). Aspirin (acetylsalicylic acid, or ASA) is one of the most commonly used medications for this purpose, particularly in the context of dual antiplatelet therapy (DAPT). However, hypersensitivity to aspirin can pose significant challenges, especially when this treatment is indispensable.

This report discusses the case of a patient with non-ST-segment elevation myocardial infarction (NSTEMI) who presented with an allergic reaction to aspirin. An aspirin desensitization protocol was successfully implemented, allowing for the initiation of DAPT following dual angioplasty. This case highlights the importance and feasibility of aspirin desensitization in ensuring optimal management of patients with aspirin allergy, in a cardiac emergency setting.

## Introduction

Antiplatelet therapy is essential for patients with acute coronary syndrome (ACS). Aspirin is considered the reference treatment due to its efficacy, availability, and low cost, particularly in developing countries [1]. It is widely recommended for the management of ST-segment elevation myocardial infarction (STEMI) and non-ST-segment elevation myocardial infarction (NSTEMI) ACS, with demonstrated survival benefits, as shown in the ISIS-2 trial [2]. The combination of aspirin with a P2Y12 receptor antagonist, known as dual antiplatelet therapy (DAPT), is especially important when a coronary stent is implanted.

Managing ACS patients with hypersensitivity to aspirin poses significant challenges. However, studies have shown that aspirin desensitization is effective for patients requiring secondary prevention of cardiovascular events [3]. Alternative strategies include the use of P2Y12 receptor inhibitors such as clopidogrel, prasugrel, or ticagrelor, which can partially replace aspirin in monotherapy. Additionally, aspirin desensitization protocols offer a safe and effective option for reintroducing aspirin, thereby enabling optimal DAPT. These approaches are crucial in ensuring the prevention of thrombotic complications in patients sensitive to aspirin.

In this case report, we describe a short and successful desensitization protocol applied to a patient who experienced a hypersensitivity reaction to aspirin, during its introduction in the setting of NSTEMI ACS.

## Case presentation

This is a 72-year-old patient with multiple cardiovascular risk factors: a 15-year history of poorly controlled type 2 diabetes treated with oral antidiabetic agents, newly diagnosed hypertension managed with angiotensin-converting enzyme (ACE inhibitors) and amlodipine besylate, and a history of dyslipidemia, treated with statins. The patient also has a smoking history but quit 15 years ago.

The patient presented to the Emergency Department with typical chest pain at rest, lasting for 30 minutes, initially neglected, and complicated by several recurrences on the day of admission.

**Figure 1:**
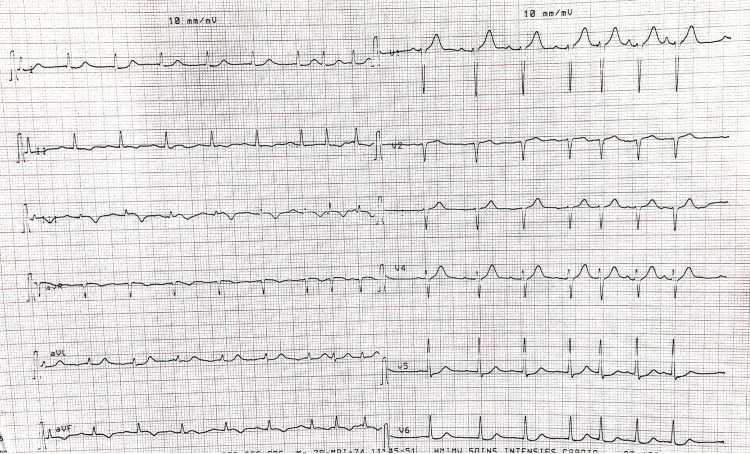
Electrocardiogram on admission An electrocardiogram shows attenuation of the R-wave in the anteroseptal territory, ST-segment depression in the lateral territory, and negative T waves in the inferior territory, consistent with myocardial ischemia.

Physical examination on admission was unremarkable. The electrocardiogram showed negative T waves in the inferior territory, along with ST-segment depression in the low lateral territory (Figure 1). Biological analysis revealed a troponin level, 200 times above the normal range. A diagnosis of high-risk NSTEMI ACS was established.

**Figure 2:**
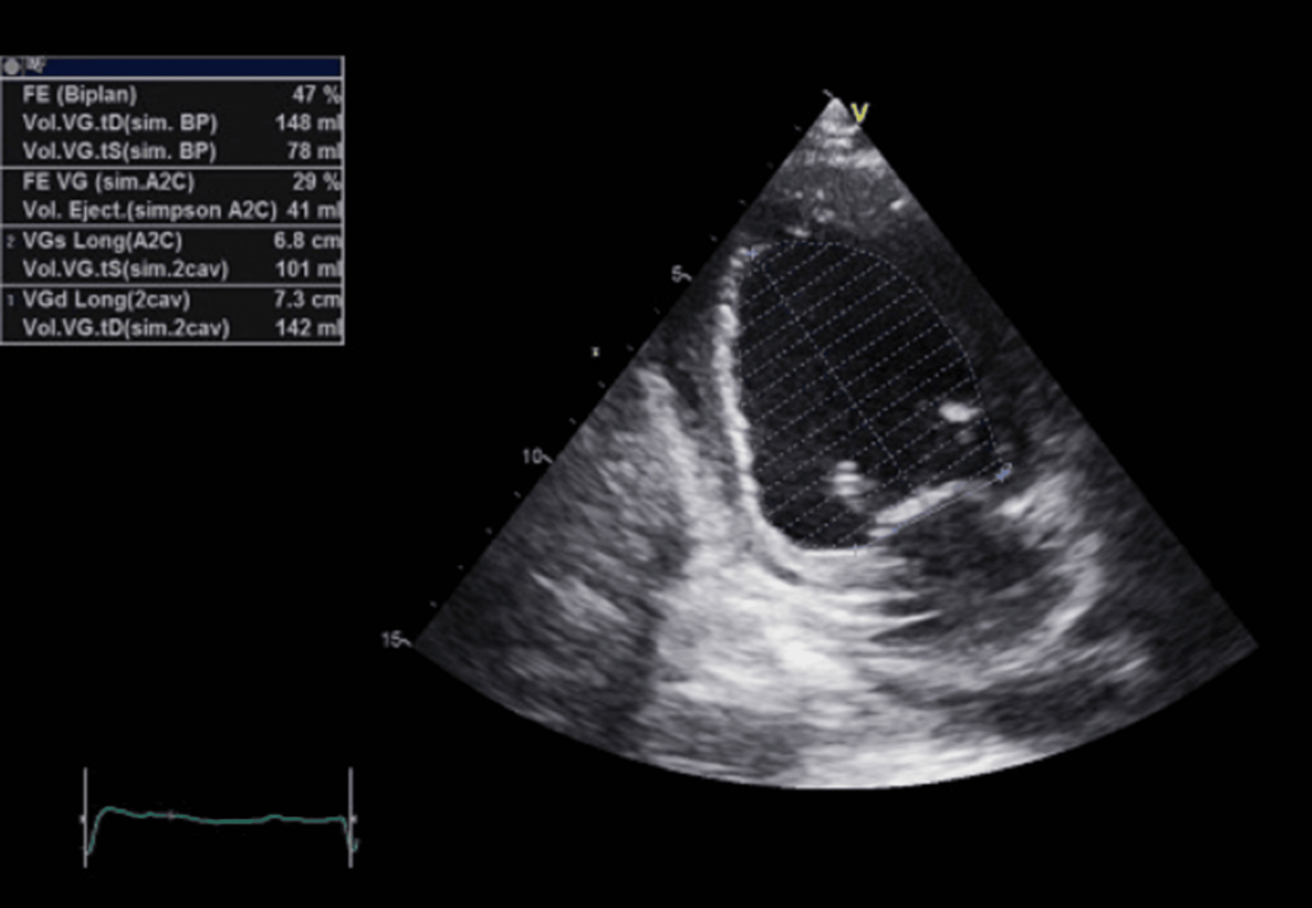
Transthoracic echocardiography A transthoracic echocardiography two-chamber shows moderately impaired systolic function and an ejection fraction of 47%.

The transthoracic echocardiography revealed a normal-sized left ventricle and a non-hypertrophied left ventricle with segmental wall motion abnormalities. Specifically, there was akinesia of the basal and middle segments of the inferoseptal wall, as well as akinesia of the apex and adjacent segments. Additionally, hypokinesia of the basal segment of the inferior wall was noted, with moderately impaired systolic function and an ejection fraction of 47%, with no visible thrombus (Figure 2).

On the day of admission, the physical examination was unremarkable. The patient was given a loading dose of antiplatelet agents, consisting of 300 mg of aspirin and 300 mg of clopidogrel administered orally, along with low-molecular-weight heparin by subcutaneous injection. One hour after administration, the patient developed a generalized pruritic rash, with skin redness on the upper trunk. He was admitted to the Cardiac Intensive Care Unit (CICU) and treated with injectable corticosteroids and antihistamines. The rash resolved completely by the second day of treatment.

Given the patient’s high ischemic risk and the allergic reaction to aspirin, a rapid aspirin desensitization procedure was implemented to ensure prolonged and effective platelet aggregation inhibition. The procedure was performed in the CICU, under close supervision with corticosteroid and antihistamine coverage.

**Figure 3:**
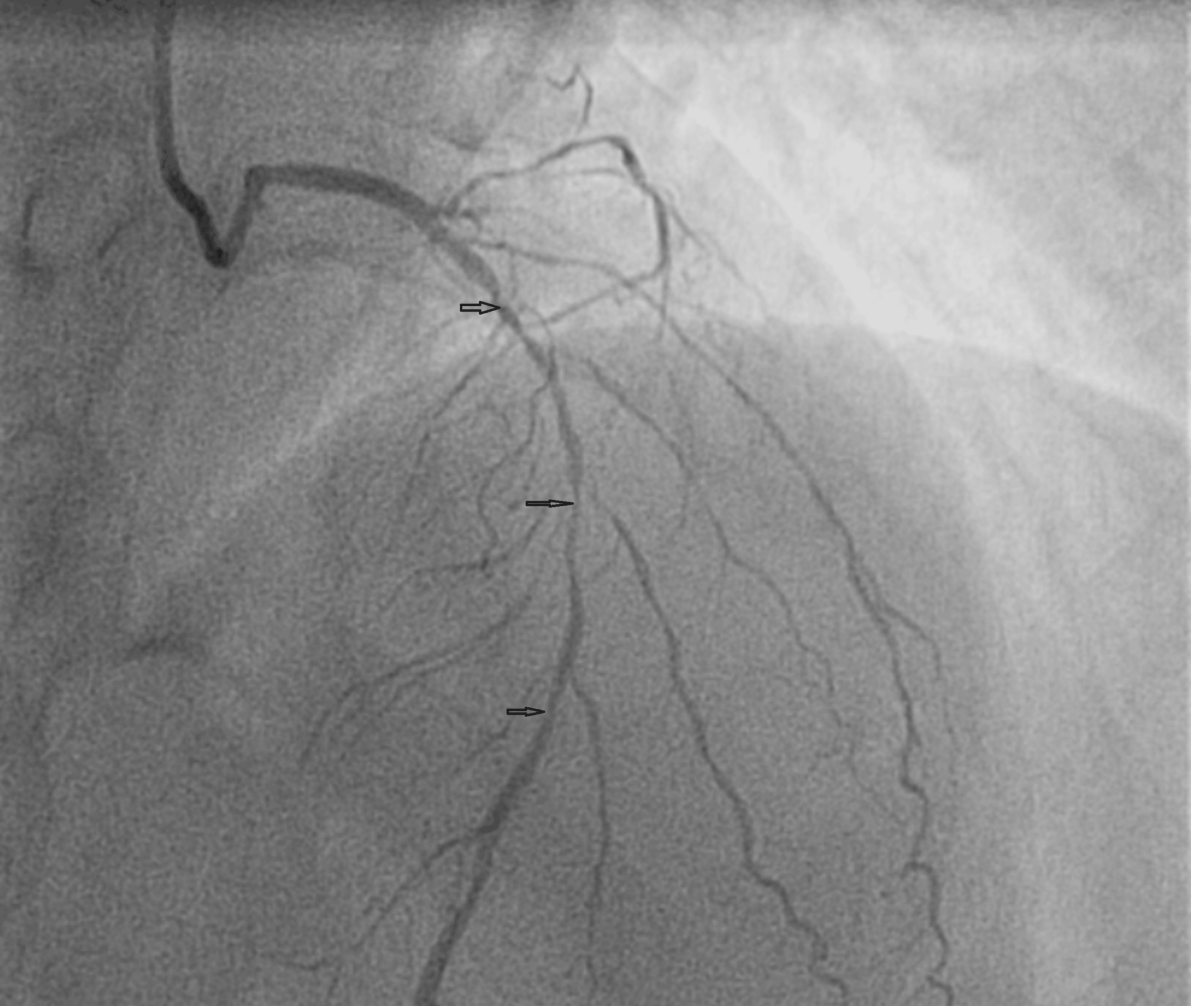
Coronary angiography Coronary angiography revealed a very long and multi-segment stenosis involving the proximal and middle segments of the LAD (black arrows). The LAD gives rise to four small-caliber diagonal branches (less than 2 mm in diameter), of which the first three exhibit significant stenoses. LAD: left anterior descending artery

The desensitization protocol spanned six hours and involved incremental doses of aspirin: 0.1 mg, 1 mg, 5 mg, 10 mg, 20 mg, 50 mg, and 75 mg, administered at one-hour intervals, reaching a cumulative dose of 160 mg. Then, the patient was transferred to the catheterization laboratory. The coronary angiography (Figure 3) was performed via the radial approach and revealed a very long and multi-segment stenosis involving the proximal and middle segments of the left anterior descending artery (LAD). The LAD gives rise to four small-caliber diagonal branches (less than 2 mm in diameter), of which the first three exhibit significant stenoses, making the lesion unsuitable for percutaneous intervention and favoring medical management, which was chosen due to the lesion's anatomical complexity and the patient's clinical stabilization. This approach aligns with current guidelines for NSTEMI management in stable patients with anatomically challenging lesions.

Then, the patient was discharged on DAPT consisting of aspirin 75 mg daily and clopidogrel 75 mg daily, and he tolerated the desensitization procedure without any significant allergic reactions.

## Discussion

Antiplatelet therapy, particularly DAPT combining aspirin and a P2Y12 receptor inhibitor, is essential in the management of ACS. This approach significantly reduces the risk of recurrent ischemic events and stent thrombosis following percutaneous coronary intervention (PCI) [4]. However, managing patients with aspirin hypersensitivity presents a significant challenge.

In our case, the patient developed an allergic reaction shortly after aspirin administration, complicating the continuation of standard treatment. The aspirin allergy was previously unknown, but its occurrence in an emergency setting necessitated rapid and effective management to prevent ischemic complications. Aspirin desensitization was therefore, chosen as the therapeutic strategy.

Rapid desensitization allowed for the safe reintroduction of aspirin without recurrence of the allergic reaction. The protocol was conducted under close supervision in the ICU, with corticosteroid and antihistamine coverage, in line with recommendations from the literature [5]. Several studies confirm the efficacy and safety of this approach, particularly in patients at high ischemic risk, where the alternative of monotherapy with a P2Y12 inhibitor may be insufficient to prevent thrombotic events [6,7].

Aspirin remains the cornerstone of antiplatelet therapy, and its combination with a P2Y12 inhibitor is strongly recommended after PCI, especially when an active stent is present [8]. The omission of aspirin could expose the patient to a heightened risk of severe cardiovascular complications. Studies have reported that aspirin desensitization achieves tolerance in over 90% of cases, enabling successful long-term reintroduction [5,9]. These findings align with our experience, as desensitization was well tolerated, and enabled secure management of the patient.

This case underscores the importance of aspirin desensitization in scenarios where aspirin allergy occurs following ACS. Having an appropriate protocol and a trained team to execute the procedure is crucial, as it offers an effective, rapid, and safe solution to maintain optimal antiplatelet therapy [10]. Additionally, it highlights the importance of meticulous follow-up after desensitization to monitor for potential allergic recurrence or treatment-related complications.

## Conclusions

The case highlights the effectiveness of rapid aspirin desensitization in a high-risk NSTEMI patient with aspirin hypersensitivity. For successful implementation of desensitization protocols in low-resource settings, it is crucial to simplify the protocol, utilize available medications such as corticosteroids and antihistamines, for supportive care, and ensure close monitoring in an ICU setting. Training medical staff and having clear, structured protocols can help mitigate challenges posed by resource limitations, ensuring safe and effective patient management.
